# Putting CATs and item banks to work: How to construct predictive and sensitive PROMIS screeners for use in ambulatory oncology

**DOI:** 10.1007/s11136-025-04015-9

**Published:** 2025-07-26

**Authors:** Benjamin D. Schalet, Michael A. Kallen, Laura M. Perry, Sofia F. Garcia, David Cella

**Affiliations:** 1 Department of Epidemiology and Data Science, Amsterdam UMC, Vrije Universiteit, Amsterdam, The Netherlands; 2 Feinberg School of Medicine, Department of Medical Social Sciences, Northwestern University, Chicago, IL USA; 3 Center for Health Outcomes, Implementation, and Community-Engaged Science (CHOICES), Tulane University School of Medicine, New Orleans, LA USA; 4 Robert H. Lurie Comprehensive Cancer Center of Northwestern University, Chicago, IL USA

**Keywords:** PROMIS, Health-related quality of life, Short-form development, Item banks, Cancer, Clinical screening

## Abstract

**Purpose:**

Incorporating PROMs into routine clinical care comes with numerous challenges, including pressure to move patients through office visits with brief, efficient assessments. The purpose of our study was to select and evaluate very short PROMIS screeners with full-bank simulations and predictive accuracy analysis.

**Methods:**

We identified candidate pairs of items for PROMIS Anxiety, Depression, Pain Interference, Fatigue, and Physical Function, on the basis of frequently selected CAT items at both normal and clinical thresholds. We then simulated full-bank responses for 10,000 patients with a T-score mean of 50 in Fatigue, Depression, Pain Interference, and Anxiety, but 45 for Physical Function. We scored candidate 2-item short-form pairs, but also evaluated a 1-item screener and a 4-item fixed CAT for comparison purposes. Clinical thresholds were set at 1 SD in the direction of worse health; diagnostic values were computed based on agreement with full-bank T-scores. We aimed for sensitivity and positive predictive values (PPV) above 0.80, considering also diversity and redundancy of content.

**Results:**

Average sensitivity of our chosen 2-item pairs across the five domains was 0.84 (range 0.80-0.89), while the average PPV was 0.82 (0.80-0.85). While the 1-item screeners for Anxiety and Depression performed poorly, they met or approached our criteria for Pain Interference, Fatigue and Physical Function.

**Conclusion:**

Five 2-item customized PROMIS forms are available for use in routine clinical care, such as distress screening in oncology. Our study provides a useful complement to psychometric criteria, helping to minimize misclassification of patients in need during screening.

**Supplementary Information:**

The online version contains supplementary material available at 10.1007/s11136-025-04015-9.

## Introduction

Patient-reported outcome measures (PROMs) allow patients to report directly on their symptoms, function, and quality of life. While initially developed for research purposes, PROMs are increasingly used in routine clinical care as a way to identify patients with concerning symptoms for potential intervention or referral [1]. The Robert H. Lurie Comprehensive Cancer Center at Northwestern University (RHLCCC), for example, has embedded symptom screening with a referral process in their ambulatory cancer clinics. The purpose of such programs is to identify, monitor and manage patients’ health needs, including referral to supportive oncology care, in accordance with the Cancer Commission on Cancer emotional distress screening standards [2–4].

Incorporating PROMs into clinical care comes with numerous challenges, including pressure to move patients through office visits with brief, efficient assessments. Brief measures, however, typically reduce the accuracy (i.e., reliability) of the assessment. Computer adaptive testing (CAT) is one possible solution, because it reduces assessment length relative to fixed-length PROMs while retaining accuracy through high reliability and tailored assessment [5]. CAT administrations, however, require readily available devices with CAT algorithms, and supporting infrastructure, at every site for every patient, which can be challenging to implement in a busy clinical setting [6].

Across various iterations, the RHLCCC screening program has included Anxiety, Depression, Pain Interference, Fatigue and Physical Function PROMIS® measures and there has been consistent feedback from clinical, administrative, and patient partners that shorter assessments are preferred [2, 3, 7]. Initially, the assessments included standard PROMIS CATs (of the above-listed domains) and checklists on psychosocial, nutritional, and informational needs. If scores were elevated, clinicians were notified of elevated scores in the electronic health record. The standard PROMIS CATs initially operational ranged from 4 to 12 items in length [8] and were completed at home via Epic MyChart, rather than in the clinic, thus limiting the pool of patients screened [2]. The motivation for our study was to support and reduce workflow burden in the routine outpatient care at RHLCCC by implementing brief, static 2-item screeners.

Is it possible that the screening purpose of the program could still function effectively with very short versions of these measures? One purpose of screening is to see whether follow-up assessment is needed. This could range from adding more questions to checking in with patients who “screen-in.” Screening allows healthcare systems to funnel precious resources (i.e., clinical care time) to patients who need them most. The question is whether a very short “low tech” PROM that is easier to implement can accurately identify patients with a high-level of need.

One way to develop and evaluate these very short PROMs is to evaluate their sensitivity and positive predictive value (PPV) [9]. Sensitivity represents the proportion of patients in need who screen positive for follow-up contact. Thus when the sensitivity value is high, it means that the screener is only missing a small number patients truly in need. The PPV represents the proportion of positive screens who are truly in need. When the PPV is high, it means most of the patients flagged by the screener for follow up are, in fact, in need. Both properties are important for a screening program where positive screens are the main focus [10, 11].

The current study describes the item selection and evaluation of five 2-item PROMIS short forms for the domains of Anxiety, Depression, Pain Interference, Fatigue, and Physical Function in the context of outpatient cancer care. Our hypotheses are that it is possible to develop 2-items PROMIS screeners with balanced content, adequate levels of sensitivity (≥ 0.80), PPV (≥ 0.80) to detect patients in need, and with adequate IRT-based reliability (≥ 0.70).

## Method

### Measurements

To evaluate screening items, we defined a patient’s true need on the basis of clinically-relevant thresholds of PROMIS full-bank scores. Full-bank scores represent scores with a very high degree of reliability across diverse health content, as detailed in PROMIS development studies [12]. The current study comprises data simulations from established item parameters and item specifications for the full banks of Anxiety v1.0, Depression v1.0 [13], Pain Interference v1.1 [14], Fatigue v1.0 [15], and Physical Function v2.0 [16]. These banks contain the following numbers of items: 29, 28, 40, 95, and 165, respectively. All PROMIS items in these banks are rated on a five-point Likert-type scale. The item parameters were originally estimated on the Graded Response Model (GRM) [17], resulting in one discrimination parameter and multiple location parameters for each item. Following the PROMIS convention, we compute and report PROMIS scores on an *expected a posteriori* (EAP) score scale, transformed to a T-score (T-score = theta✕10 + 50) [18], where 50 represents an estimated mean of the US general population and 10 its standard deviation. For all banks except Physical Function, higher scores indicate worse health relative to the mean T-score of 50. For example, a T-score of 60 on Fatigue indicates that the respondent experiences more fatigue than the average of the general population of 50. For Physical Function, the opposite is true, such that lower scores (such as 35) indicate poorer functioning than the mean of 50. In order to create a dataset of simulated patients with PROMIS responses, we needed to load the official PROMIS item parameters of the full-banks into the CAT simulation program (parameters are available for psychometric work by permission from the PROMIS Health Organization via enquiry at www.healthmeasures.net).

### Methods and analysis

*Identification of candidate 2-item pairs via CAT simulations.* Given the size of the banks (28–165 items), it would be unwieldy to calculate sensitivity and PPV values for every possible very short (2-item) pair. One approach for initial short-form item-selection is to apply a CAT-based algorithm, a procedure originally developed in psychology [19, 20], later applied and evaluated in the context of PROMIS short-forms development across multiple health domains [21–23]. The CAT algorithm selects items based on the maximum IRT-based information weighted by the posterior distribution across the simulated population of the domain (MPWI) [24]. This algorithm selects successive items by dynamically adjusting the weighting of information according to a (simulated) person’s previous responses, reflecting varying levels of health. Thus, by identifying frequently selected items by this CAT algorithm, we narrowed down the items needed for the screener. While other psychometric procedures are available to select high-information items from an item bank [23, 25], we were partially motivated to use CAT to select screener items given the historical use of CAT in selected RHLCCC clinics, and the potential future need to merge the new screener and older CAT data.

To do so, we simulated a dataset of patient PROMIS scores, reflecting the score distribution of an outpatient oncology center. On the basis of current oncology literature with PROMs [2, 3, 26], we simulated a set of full-bank responses for a sample of 10,000 patients with a T-score mean of 50 in Fatigue, Depression, Pain Interference, and Anxiety, but 45 for Physical Function, and an SD of 10 for all domains. Next, we conducted post-hoc CAT simulations on these samples, which allowed us to identify items that were chosen as the 1st, 2nd or 3rd item by the CAT in at least 10% of the simulated patients. From this pool of items, we made 2-item pairs. In each pair, we included the 1st item selected by the CAT, which has the highest information at the estimated mean of the simulated oncology center sample. We did this for the sake of partial consistency with standard PROMIS CAT and fixed-forms administrations, which also include this item [22]. Pairing this item with the 2nd and 3rd CAT items lead to five pairs for Anxiety, Depression, and Physical Function, and six pairs for Fatigue and Pain Interference.

For comparison purposes, and to ensure we did not overlook any important items, we also applied a simpler (non-CAT) psychometric procedure for identifying candidate screener items. We identified the two most informative items at the clinical threshold point for each domain and, additionally, by information weighted by a standard normal T-score distribution around the clinical thresholds (thresholds are defined below). This approach led us to identify two items per domain, but all 10 items had already been identified by the CAT procedure. (Interested users can see these “high information” items in Table S1).

Finally, we inspected the 5–6 item pairs for content overlap. Across the 5 domains, 10 items were removed because of similarity in content within the domain, 2 items were removed due to the narrowness of the item concept, and 1 was removed because of overlap with another domain. This process resulted in three pairs to evaluate for all domains, except for Pain Interference, which was left with two pairs. Supplemental Table S1 provides the details.

*Evaluation of 2-item pairs.* Next, we evaluated the pairs of items as short-forms based on the sensitivity and PPV in relation to the full-bank clinical thresholds. Because PPVs are dependent upon the prevalence of the target disease (operationalized in our case as above the full-bank threshold), we used the simulated dataset specified above, reflecting the score distribution of an oncology outpatient center. We then identified reasonable boundaries for clinically-relevant symptoms, on the basis of PROMIS standard setting studies [27, 28] and predictive studies with cancer patients [29]. We set the clinical threshold at 1 SD in the direction of poorer health relative to the mean score. This means that a T-score of 60 for Anxiety, Depression, Pain Interference, and Fatigue represents a score that is 1 SD (10 T-score points) in the direction of “worse” health relative to the general population mean. For Physical Function, we used a T-score of 35 as the clinical threshold. Heretofore, we will call this boundary score the “clinical threshold.”

From here, we scored the candidate 2-item short-form pairs. We also evaluated a 1-item screener and a 4-item fixed-length CAT for comparison purposes. We used the first item selected by the CAT algorithm as the 1-item screener. To make the final selections, diversity and any redundancy of content were also considered.

To calculate our predictive values, we set the screener cut point to be the same as the clinical thresholds defined above (35 for Physical Function and 60 for the remaining domains). Patients above these clinical thresholds represent approximately 15% of an outpatient cancer sample [2, 3]. In the evaluation, we sought to identify screeners with a sensitivity and PPV of 0.80 or greater. The sensitivity target is in line with acceptable performance of depression screeners [30–32]. Our thresholds align with classification as “good utility” on the Clinical Utility Index (CUI+), which is the product of sensitivity and PPV. CUI + values are classified as excellent utility ≥ 0.81, good at ≥ 0.64, satisfactory utility ≥ 0.49, and poor utility ≤ 0.49 [11, 33]. We also chose a target 0.80 for PPV on practical grounds: no more than 20% of the positive screens should be false (i.e., relatively healthy, and therefore not needing follow-up contact).

We conducted our analyses in R [34] with the program Firestar (v1.3.2) [35], which is a CAT simulation program for polytomous IRT models that has been customized for use with PROMIS item banks. Firestar generates R code, given various settings controlled by the user (e.g., item selection method, scoring algorithm, stopping rules). Consistent with PROMIS usage, the item selection proceeded with MPWI [24]. Sensitivity and PPV were calculated with the aid of the R package yardstick [36].

## Results

**Fig. 1:**
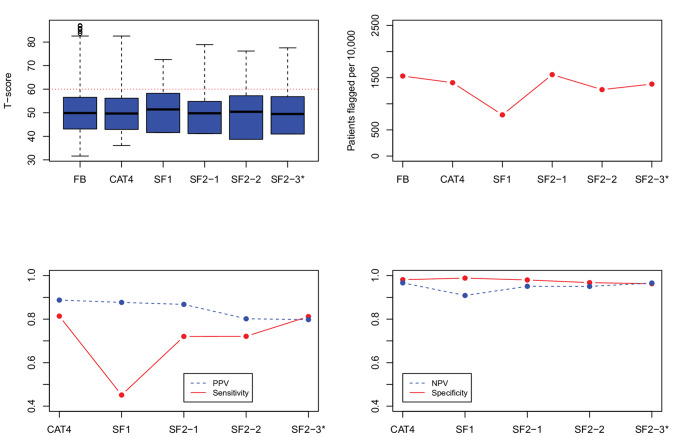
PROMIS Anxiety screener development results, showing box plots and simulated patients flagged on the top panels, and predictive values on the bottom panels. FB = full-bank T-score serving as the gold standard score. SF1 = one item short form. CAT4 = four item fixed-length CAT score. SF2_1, SF2_2, and SF2_3 indicate the candidate 2-item pairs. SF2_3* is the candidate pair we selected. PROMIS IDs are: SF2_1 = EDANX53, EDANX40; SF2_2 = EDANX53, EDANX54; SF2_3 = EDANX53, EDANX41

**Fig. 2:**
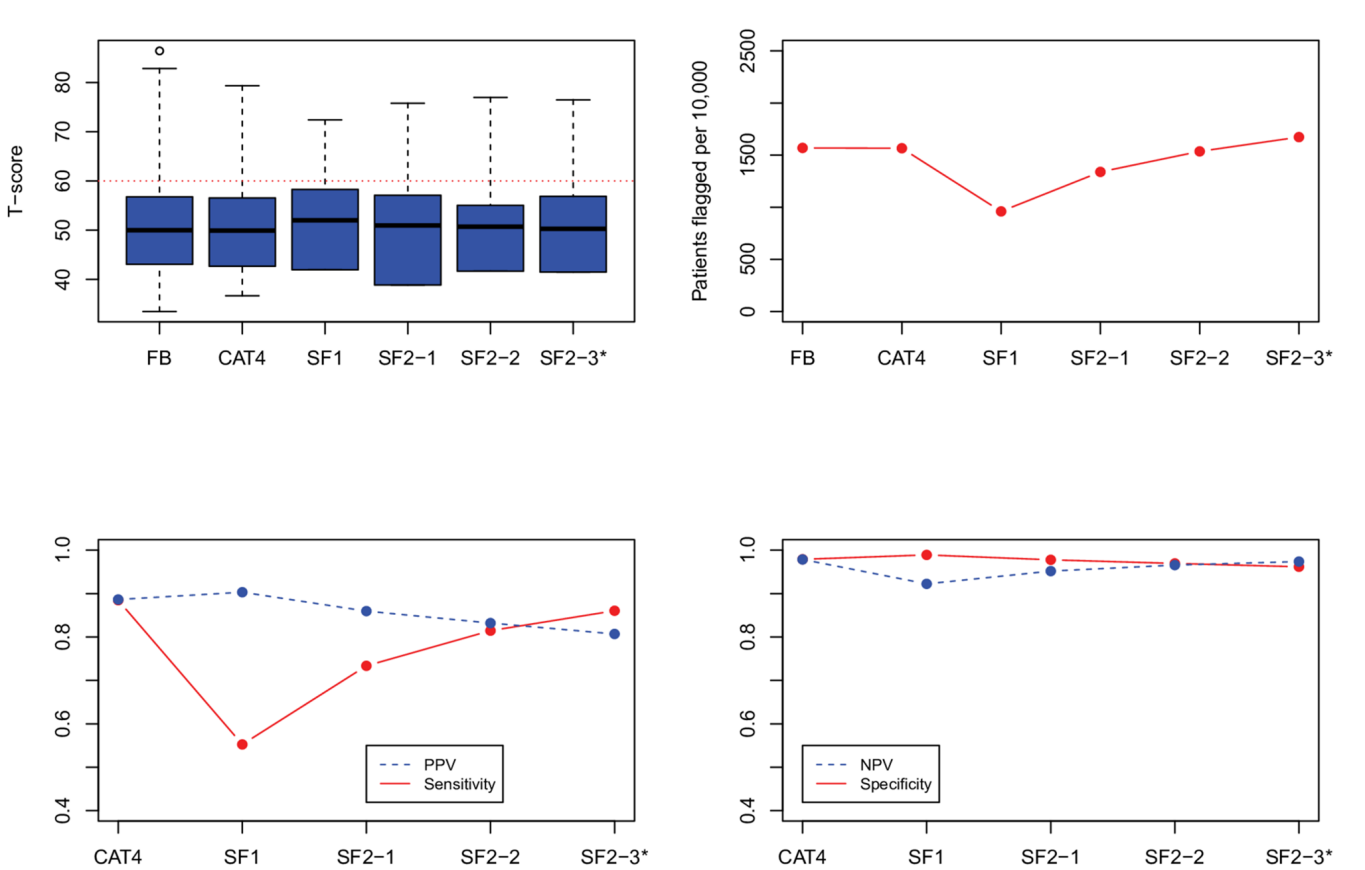
PROMIS Depression screener results, showing box plots and simulated patients flagged on the top panels, and predictive values on the bottom panels. FB = full-bank T-score serving as the gold standard score. SF1 = one item short form. CAT4 = four item fixed-length CAT score. SF2_1, SF2_2, and SF2_3 indicate the candidate 2-item pairs. SF2_3* is the candidate pair we selected. PROMIS IDs are: SF2_1 = EDDEP29, EDDEP36; SF2_2 = EDDEP29, EDDEP41; SF2_3 = EDDEP29, EDDEP04

**Fig. 3:**
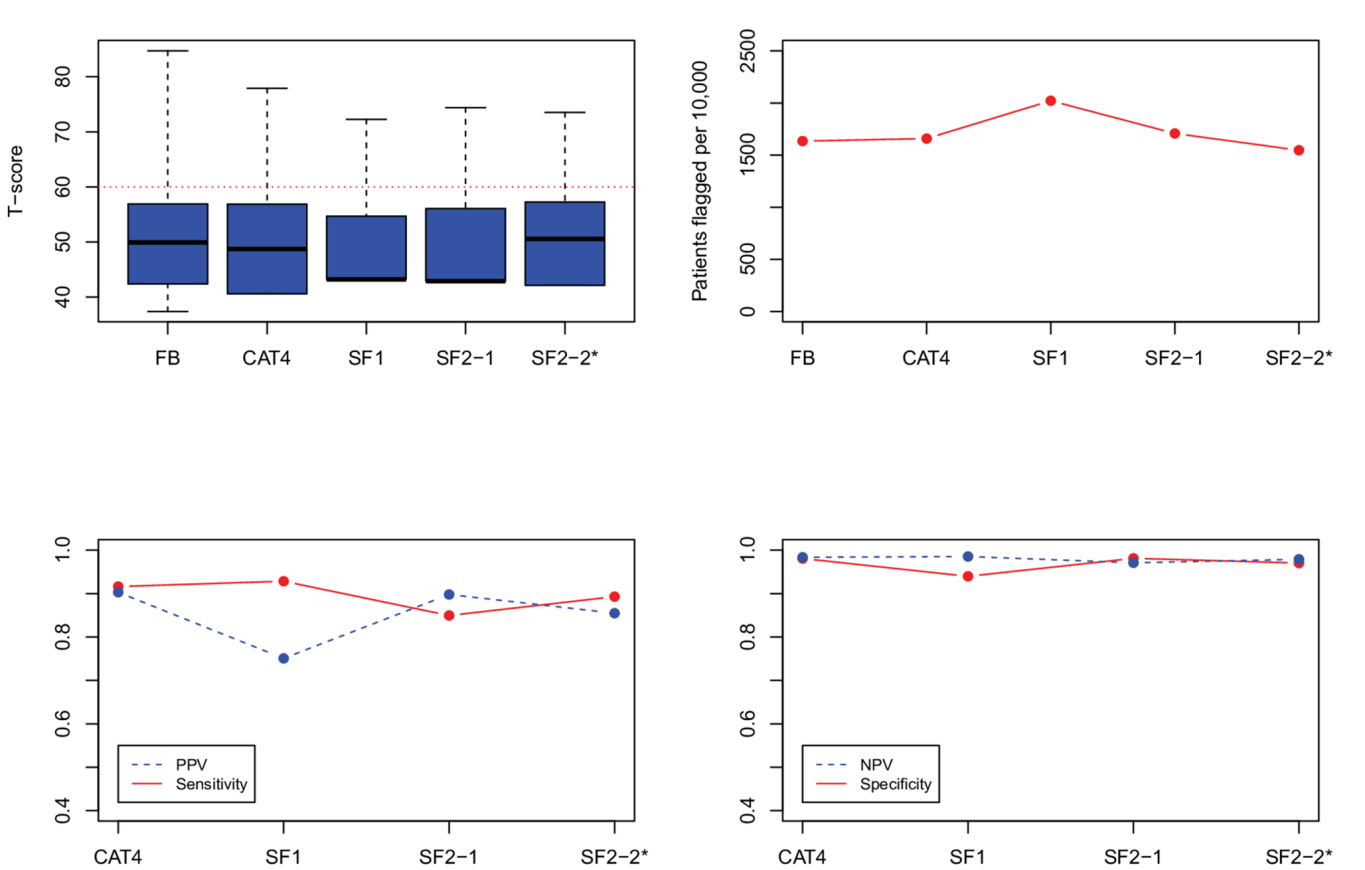
PROMIS Pain Interference screener results, showing box plots and simulated patients flagged on the top panels, and predictive values on the bottom panels. FB = full-bank T-score serving as the gold standard score. SF1 = one item short form. CAT4 = four item fixed-length CAT score. SF2_1 and SF2_2 indicate the candidate 2-item pairs. SF2_2* is the candidate pair we selected. PROMIS IDs are: SF2_1 = PAININ9, PAININ31; SF2_2 = PAININ9, PAININ10

**Fig. 4:**
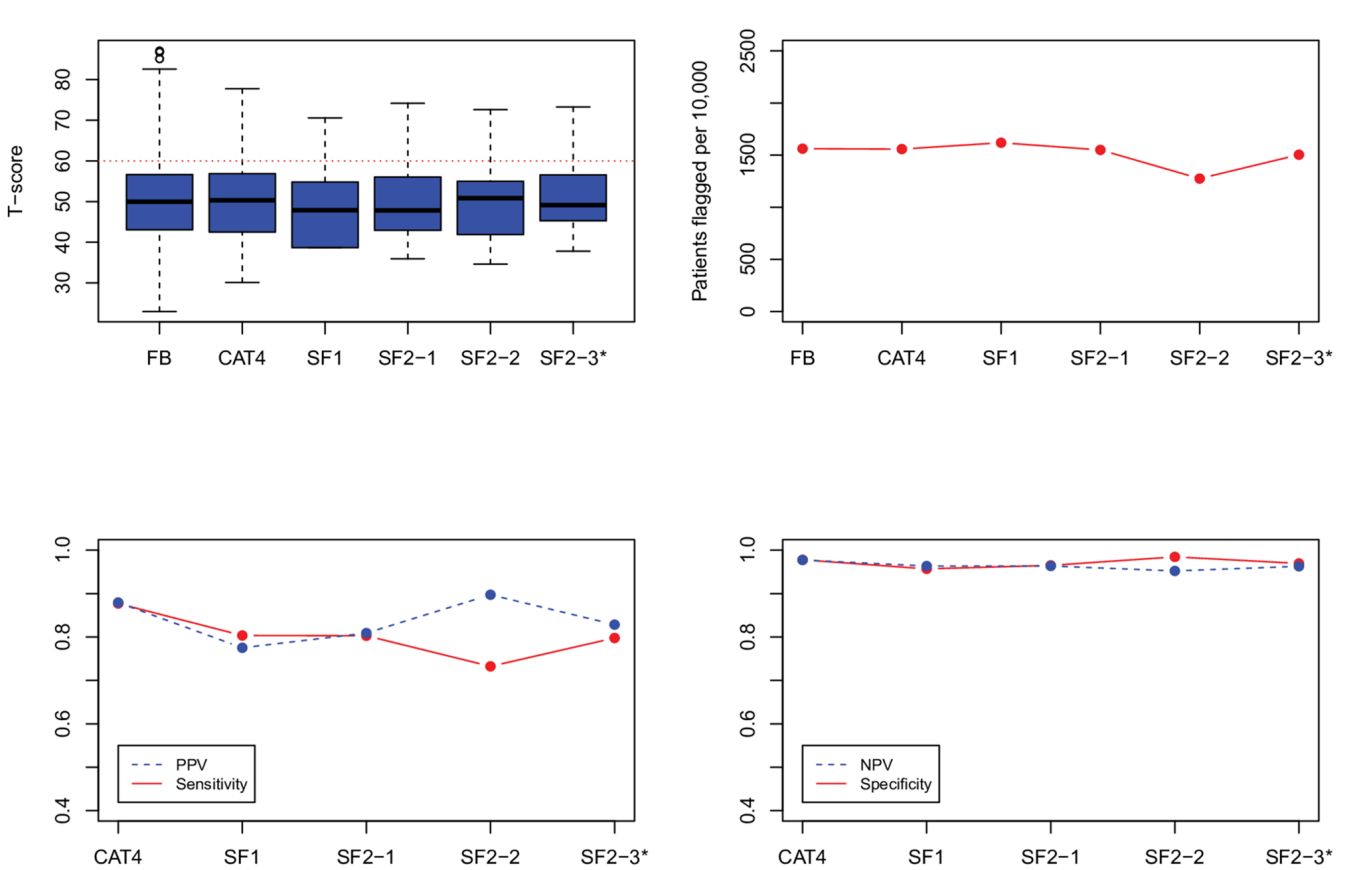
PROMIS Fatigue screener results, showing box plots and simulated patients flagged on the top panels, and predictive values on the bottom panels. FB = full-bank T-score serving as the gold standard score. SF1 = one item short form. CAT4 = four item fixed-length CAT score. SF2_1, SF2_2, and SF2_3 indicate the candidate 2-item pairs. SF2_3* is the candidate pair we selected. PROMIS IDs are: SF2_1 = FATIMP3, FATEXP40; SF2_2 = FATIMP3, HI7; SF2_3 = FATIMP3, AN3

**Fig. 5:**
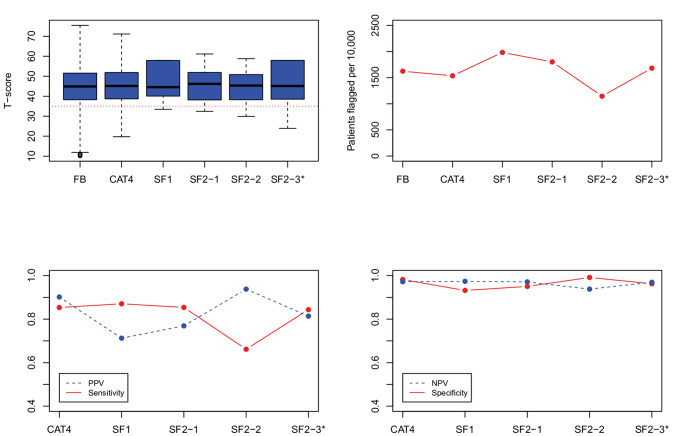
PROMIS Physical Function screener results, showing box plots and simulated patients flagged on the top panels, and predictive values on the bottom panels. FB = full-bank T-score serving as the gold standard score. SF1 = one item short form. CAT4 = four item fixed-length CAT score. SF2_1, SF2_2, and SF2_3 indicate the candidate 2-item pairs. SF2_3* is the candidate pair we selected. PROMIS IDs are: SF2_1 = PFC12, PFB7; SF2_2 = PFC12, PFC11; SF2_3 = PFC12, PFB13

***Evaluation of candidate SF item-pairs.*** Figs. 1, 2, 3, 4 and 5 show the results for the five domains; each figure has 4 panels plotting the 2-item candidate SFs along with the 4-item fixed-length CAT, expected to perform better, and the 1-item SF, expected to perform worse. The candidate pair we selected as final is always the last SF listed to the right and is indicated with an asterisk (“*”). Though it is not our focus, we also report on the specificity and negative predictive value (NPV), which were uniformly high (> 0.90). (In these figures, we connected the dots with lines to show the connection between the property being plotted for different forms; the lines do not mean to imply a time-series.)

*Anxiety.* The top-left panel of Fig. 1 shows that the three candidate 2-item pairs have a similar range, albeit naturally smaller than the full-bank and 4-item fixed-length CAT. The ceiling of the three 2-item SFs extends well past the clinical threshold. The top right panel shows that all 3 forms flag around 15% of the simulated patients; the best SF should flag the same proportion of patients as the full-bank; the SF2-3 comes closest. The bottom left panel shows that the sensitivity and PPV of SF2-3 is more favorable than the others (with both values around 0.80). SF1 performed poorly in terms of sensitivity (< 0.6), although the PPV was high.

*Depression.* Figure 2 (Depression) shows a similar pattern as Fig. 1, except that three 2-item SFs differ in sensitivity, reciprocally relative to PPV. We selected SF2-3 based on higher sensitivity at a slight cost to PPV.

*Pain Interference.* Figure 3 (Pain Interference) shows a somewhat different pattern in that the sensitivity and PPV of both 2-item SFs are between 0.8 and 0.9. We choose SF2-2 based on slightly higher sensitivity. The 1-item SF flagged more patients than the full-bank score (20% vs. 16%), and consequently the PPV was slightly lower as well (0.75), compared to the chosen SF2-2 (0.85).

*Fatigue.* Figure 4 (Fatigue) shows a remarkable similarity in the number of patients flagged by each form. SF2-2, however, flagged fewer patients and also shows lower sensitivity relative to the other forms. SF2-1 and SF2-3 shows similar sensitivities and PPV values; SF2-3 was chosen based on preferable content. The performance of the 1-item SF was similar to the SF2-3, showing a sensitivity of 0.80 and PPV of 0.78.

*Physical Function*. Figure 5 (Physical Function), top left panel, shows that the SF2-1 and SF2-2 have minimum values close to the clinical threshold. They also deviate more than SF2-3 from the numbers of simulated patients flagged by the full bank. The bottom left panel shows that the sensitivity of SF2-2 was relatively poor (0.66). The PPV of the SF2-3 was slightly higher than the SF2-1, while the sensitivities of these 2 forms were similar. Taken together, we chose SF2-3 on this basis and a preference for the item content. (Interested readers can see results for additional clinical thresholds as Supplementary Figs. 1–5 S.)

*Summary.* Table 1 summarizes the results and information for the selected 2-item screeners we selected for each domain. It provides the sensitivity, PPV, and mean IRT model reliabilities for the final 2-item SFs, along with the PROMIS item IDs and item stems. The average sensitivity across the five domains was 0.84, while the average PPV was 0.82. The mean IRT reliabilities of the 2-item forms are 0.75 or higher, reflecting the high inter-correlation of each item pair in the calibrated IRT model.

**Table 1:** Simulated means and thresholds, sensitivity, positive predictive power, and reliability for 2-item screeners based on PROMIS

	Anxiety	Depression	Pain Interference	Fatigue	Physical Function
Simulated Sample Mean (SD)	50	50	50	50	45
Clinical Threshold	≥ 60	≥ 60	≥ 60	≥ 60	≤ 35
Sensitivity	0.81	0.86	0.89	0.80	0.84
PPV	0.80	0.81	0.85	0.83	0.81
CUI (+)	0.65	0.69	0.76	0.66	0.69
CUI (-)	0.93	0.94	0.93	0.95	0.93
Mean IRT model reliability	0.75	0.76	0.77	0.85	0.79
Item #1 PROMIS ID	EDANX53	EDDEP29	PAININ9	FATIMP3	PFC12
Item #1 Stem	*I felt uneasy*	*I felt depressed*	*How much did pain interfere with your day to day activities?*	*How often did you have to push yourself to get things done because of your fatigue?*	*Does your health now limit you in doing two hours of physical labor?*
Item #2 PROMIS ID	EDANX41	EDDEP04	PAININ10	AN3	PFB13
Item #2 Stem	*My worries overwhelmed me*	*I felt worthless*	*How much did pain interfere with your enjoyment of recreational activities?*	*I have trouble* *starting* *things because I am tired*	*Are you able to carry a shopping bag or briefcase?*

PPV = Positive Predictive Value. CUI = Clinical Utility Index. CUI(+) represents the Sensitivity✕PPV, while CUI(-) represents Specificity✕Negative Predictive Value. Values greater than 0.64 are considered good, while those above 0.81 are considered excellent [10]

While the 1-item screeners for Anxiety and Depression performed poorly (sensitivity < 0.6), they actually met or came close to our criteria for Pain Interference, Fatigue and Physical Function. All three met our threshold for sensitivity, and were slightly below the threshold in PPV (0.78, 0.75, and 0.71, respectively). The 1-item Pain Interference screener met the CUI(+) criteria also exceeded the cut-off for “good utility” with 0.70. The 4-item fixed-length CAT performed better across all domains with respect to sensitivity and PPV than the 2- and 1-item forms, as expected.

## Discussion

We customized 2-item PROMIS short-forms to support routine symptom screening in outpatient cancer clinics. Applying multiple methods, we identified 2-item screeners for PROMIS Anxiety, Depression, Pain Interference, Fatigue, and Physical Function that demonstrated reasonably high sensitivity (mean of 0.84) and PPV (mean of 0.82) to clinically relevant ranges. Our method of selecting and evaluating items is novel because it focusses on the performance of the screener, rather than exclusively on the psychometric properties based on inter-item correlations. A strength of this study, therefore, is the inclusion of performance criteria included PPV (proportion of patients flagged who are truly in need) and sensitivity (proportion of patients truly in need who are also flagged). We furthermore defined true patient need based on large full-bank PRO clinical threshold scores. This is necessary given the general difficulty of identifying a PRO gold standard for “patients truly in need” across the different health domains [37]. The availability of full-bank IRT parameters allowed us to simulate scores, based on reasonable assumptions on the distribution of PROM scores in oncology screening clinics. Borrowing from previous methodological studies on item selection for SFs [22, 38], we applied CAT algorithms and available PROMIS IRT parameters to select informative candidate items at the mean and clinical thresholds, given the oncology patient distribution. (Information on custom PROMIS forms and scoring can be obtained from the authors or at www.healthmeasures.net.)

As expected, the 4-item fixed-length CAT produced the highest sensitivity and PPV values among all forms, while the 1-item screener naturally performed less well, though this varied markedly by domain. For Anxiety and Depression, the sensitivity of the 1-item SF was clearly quite low (~ 0.5), but for Pain Interference and Fatigue sensitivity remained high, while the PPV values were just below our 0.80 threshold. The pain screener even showed a CU1 + value that exceeded the “good utility” threshold. We may conclude that while 2-item screeners are slightly better, the 1-item screener can be a reasonable choice for Fatigue and Pain Interference in contexts where a minimum set of questions need to be asked.

Our analyses highlight the value of looking at both psychometric properties and predictive performance when selecting items for short-forms. Effective screeners must have scores that extend into the clinical range of interest, and in the case of an IRT-calibrated item bank, researchers can attend to this by selecting high information items in the clinical range. It is important to examine the predictive values in light of a gold standard and a screener goal (e.g., to minimize false negatives). For example, for Depression and Physical Function domains, the 3 candidate SFs differed considerably in terms of sensitivity and PPV. These predictive values are also a function of the distribution of scores in the screening population, the clinical threshold of the gold standard, and the clinical threshold of the screener. Finally, a predictive values analysis is helpful, because PRO scores can have idiosyncratic scoring distributions that may not be obvious from item information curves. These idiosyncrasies could result in deviations in the number of patients flagged relative to the gold standard, resulting in better or poorer screener performance.

Throughout our procedure, we balanced statistical properties with clinical content considerations. This is important not only to avoid redundant item content, but also to avoid a potentially “jarring” assessment experience. That is, screeners providing the most psychometric information at threshold will reflect “sicker” content. For example, the two most informative Depression items at threshold of 60 ask if patients feel “worthless” and “hopeless.” While these items might be excellent for identifying patients in need, they are not suited to most patients and could induce a feeling in some patients of their experiences not being represented by the healthcare system [39].

Our final 2-item forms showed some overlap, but also differences with existing PROMIS short-forms. For Anxiety and Depression, our 2-item pairs were also included in the PROMIS 29 Profile v2.0 [22], but for Pain Interference and Fatigue only 1 item overlapped. For Physical Function neither of our items overlapped with the PROMIS 29 profile. The PROMIS 57 v2.0 Profile included all of our items, except for 1 Physical Function item. We describe the level of overlap, because it may inform score comparisons across studies with different PROMIS forms and data aggregation efforts. (The PROMIS item IDs are listed in Table 1.)

Our study was conducted in parallel with a recently published paper on 2-item screeners for the PROMIS profile domains [25]. The study by Edelen et al. selected 2-item SFs on the basis of previous inclusion in short-forms, IRT properties, content balancing, and stakeholder input. For all domains except Physical Function, one item in each of their screener pair corresponded to an item in our final SFs. For Depression and Pain Interference, the 2 final items selected by Edelen et al. are identical to our candidate pairs SF2-2 and SF2-1, respectively. Both performed very similarly to our final forms (see Figs. 2 and 3). For Physical Function, however, the SF from Edelen et al. did not include any items we selected or tested. We note that the two items were entirely different in content (climbing stairs and going for a walk), and showed somewhat lower discrimination parameter values than the 2 final items we selected (~ 0.5). While IRT-based criteria lead both study teams to select nearly the same items for 4 out of the 5 domains, for Physical Function the differences in procedure (such as the inclusion by Edelen of stakeholders in narrowing down candidate items) may have caused different final item selection.

We should note some limitations and alternatives to our methods. First, our initial procedure for selected suitable screener items relied on the MPWI-CAT algorithm, deployed as a standard with PROMIS [8; 24] and used previously to assist with short-form development [21–23]. Our CAT strategy was not only motivated by its previous application in the PROMIS short-form literature, but also due to our wish for comparability with actual CAT item-level data, given that the previous screening program at RLHCCC was based on CATs [40]. Alternative psychometric methods for item selection could have been applied instead, such as those described by Choi et al. (2010) [23], including the ranking of items based information weighted by the population around the clinical threshold. Using the later strategy exclusively may have led to slightly different item selections, depending upon content decisions (see Table S1). Users wishing to avoid the complexity of CAT simulations could calculate such a weighted information ranking once and determine preferred items from there. As a final alternative, we could have selected item pairs purely based on superior predicted performance (i.e., CUI+) coupled with content considerations, but ignoring differences in psychometric item information. However, this would have required an unwieldy number of tests, given the bank sizes (28–165 items), and would have risked minimal item overlap with previous screening data collected via CAT. Nevertheless, other researchers may wish to adopt this strategy if such setting-specific considerations are not a factor and if lower internal consistency of the resulting screener is an acceptable outcome.

Secondly, our study relied on simulated patient data. Though our simulations were driven by item parameters originally estimated from large samples of actual patient data [12], it will be useful to replicate our study with observed data. Third, our gold-standard was defined by the full-bank PROMIS score and clinical thresholds supported by research [27]; thus, we did not (and could not) define the gold-standard as a clinical diagnosis of, for example, cancer-related fatigue or major depression. Nevertheless, our procedure was consistent with the purpose of the Cancer Commission screening standards, which was to serve patients with holistic psychosocial distress problems, which may not reach the severity of a specific diagnosis [4].

Our study was designed to meet the needs of oncology outpatient clinics, using known findings for population means and clinically-relevant thresholds supported by research in the simulations [2; 3; 26]. However, with the exception of Physical Function, the PROMIS T-score means in the simulation reflect general population means. Given the generic nature of PROMIS items, our results are likely to be applicable to other clinical care settings where the score distributions are similar. Nevertheless, some clinical settings (such as pain clinics) may reflect specific patient populations with more elevated means and clinical threshold scores. Applying our methods to such settings could have altered the selection of screener items and resulting predictive values. In general, a more extreme clinical threshold might lead to the selection of different items and/or predictive values. (Interested readers can see the Supplemental Figs. 1–5 S for results at a variety of thresholds.) If a screener needs to select more extreme, that is rarer, cases (e.g., T-score > 70 on Fatigue), then it is better to administer a longer instrument, such as the 4-item SF or CAT.

A final potential limitation is that sensitivity and PPV values of 0.80, while reasonably high, also imply false negative and false discovery rates of 20%. Our results suggests that longer measures could further increase sensitivity and PPV, but additional investigation would be needed to confirm this and evaluate the gains attributable to SF length and longer CATs. However, regardless of the improvements beyond 0.80, this would need to be weighed against the benefit of the time savings in routine care by administering very short screeners, requiring approximately 2 minutes of patient time for 10 items total [41].

## Electronic supplementary material

Below is the link to the electronic supplementary material.

Supplementary Material 1

Supplementary Material 2

## Declarations

### Conficts of interest

B.D. Schalet and D. Cella are board members of the PROMIS Health Organization. The other authors have no conficts of interest to declare that are relevant to the content of this article.
