# Supplementary material for: Putting CATs and item banks to work: How to construct predictive and sensitive PROMIS screeners for use in ambulatory oncology
Source: Qual Life Res. 2025 Jul 26;34(10):2775–85. doi: 10.1007/s11136-025-04015-9 (PMC12535503; doi:10.1007/s11136-025-04015-9)

**Supplement Figures: Sensitivity and Predictive Values for PROMIS screeners by Multiple Clinical Thresholds**

Figure 1S. PROMIS Anxiety screener development results, showing predictive values for multiple clinical thresholds SF1 = one item short form. CAT4 = four item fixed CAT score. SF2_1, SF2_2, and SF2_3 indicate the candidate 2-item pairs. SF2_3* is the candidate pair we selected. PROMIS IDs are: SF2_1 = EDANX53, EDANX40; SF2_2 = EDANX53, EDANX54; SF2_3 = EDANX53, EDANX41.


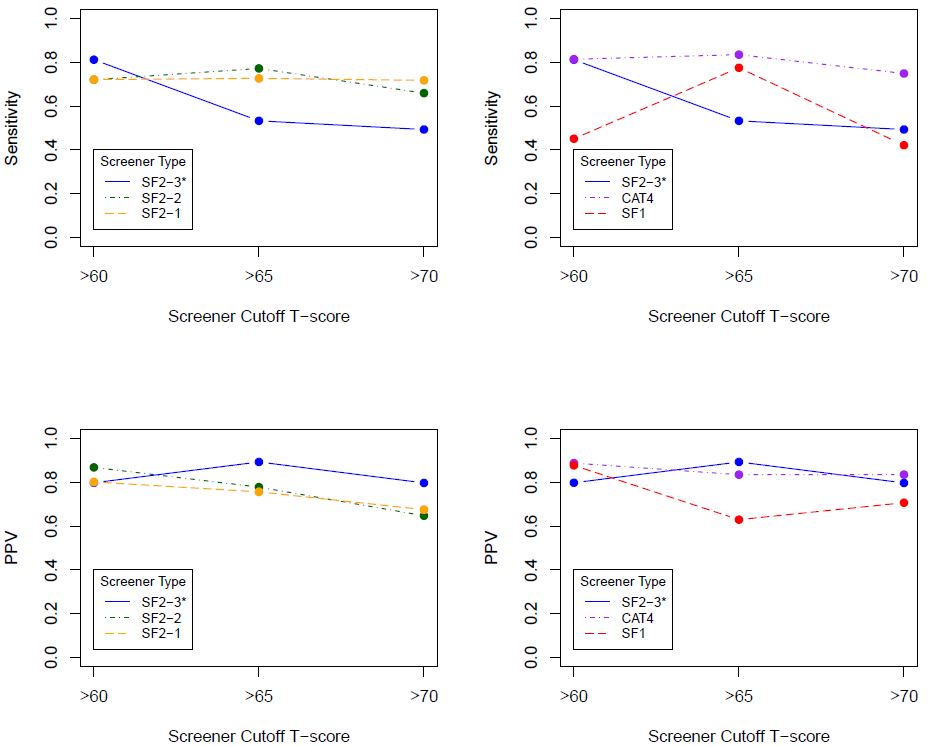


Figure 2S. PROMIS Depression screener results, showing predictive values for multiple clinical thresholds SF1 = one item short form. CAT4 = four item fixed CAT score. SF2_1, SF2_2, and SF2_3 indicate the candidate 2-item pairs. SF2_3* is the candidate pair we selected. PROMIS IDs are: SF2_1 = EDDEP29, EDDEP36; SF2_2 = EDDEP29, EDDEP41; SF2_3 = EDDEP29, EDDEP04.


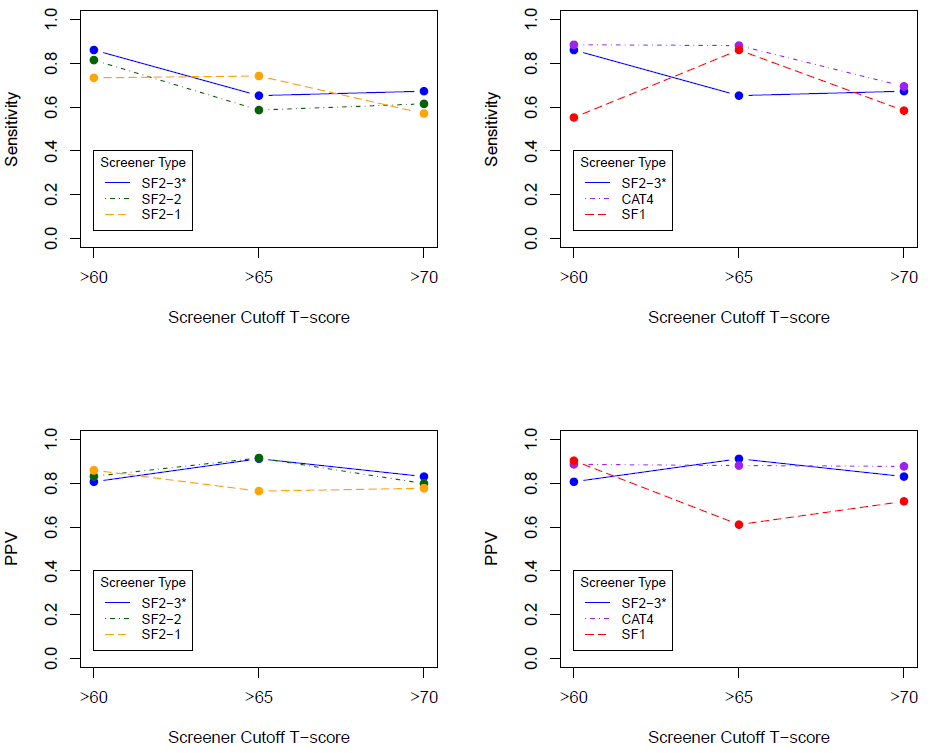


Figure 3S. PROMIS Pain Interference screener results, showing predictive values for multiple clinical thresholds SF1 = one item short form. CAT4 = four item fixed CAT score. SF2_1 and SF2_2 indicate the candidate 2-item pairs. SF2_2* is the candidate pair we selected. PROMIS IDs are: SF2_1 = PAININ9, PAININ31; SF2_2 = PAININ9, PAININ10.


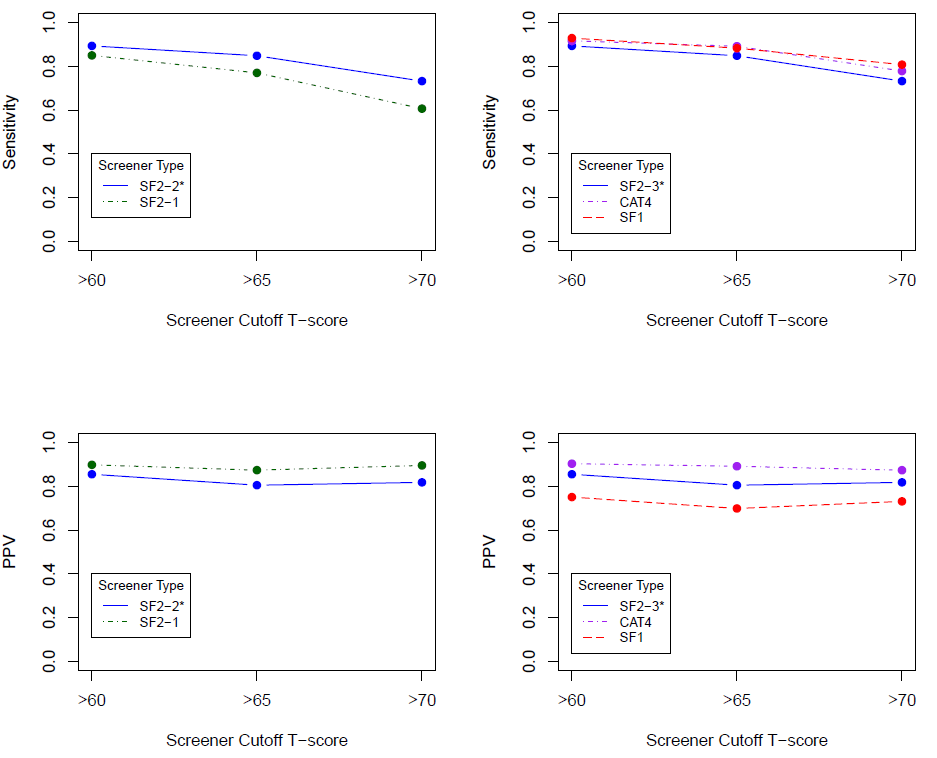


Figure 4S. PROMIS Fatigue screener results, showing predictive values for multiple clinical thresholds SF1 = one item short form. CAT4 = four item fixed CAT score. SF2_1, SF2_2, and SF2_3 indicate the candidate 2-item pairs. SF2_3* is the candidate pair we selected. PROMIS IDs are: SF2_1 = FATIMP3, FATEXP40; SF2_2 = FATIMP3, HI7; SF2_3 = FATIMP3, AN3.


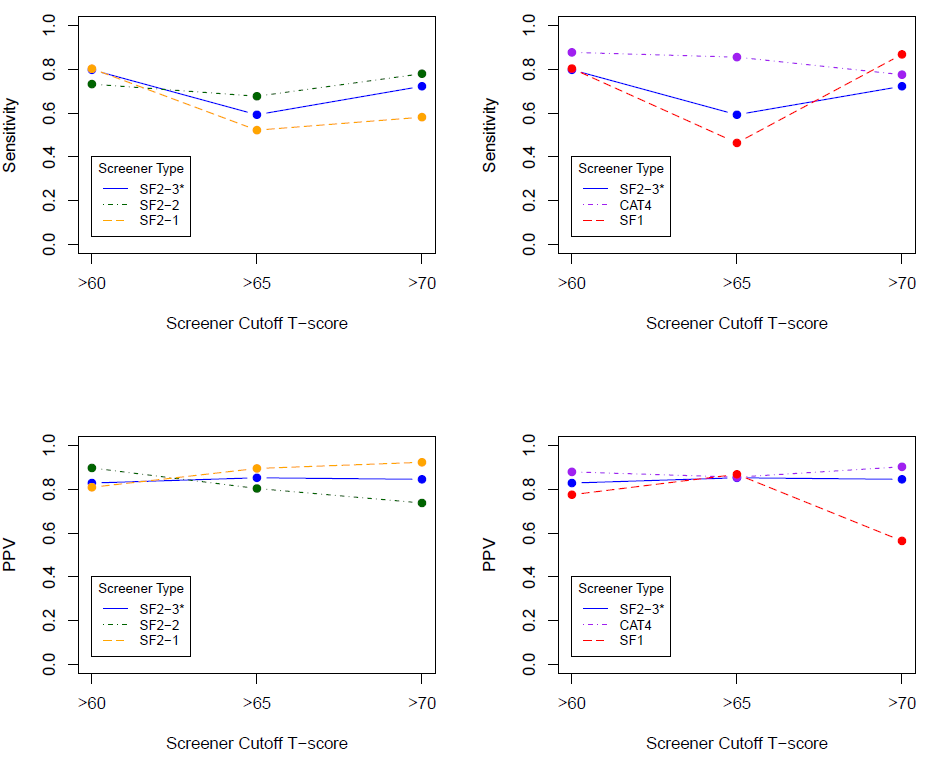


Figure 5S. PROMIS Physical Function screener results, showing predictive values for multiple clinical thresholds. SF1 = one item short form. CAT4 = four item fixed CAT score. SF2_1, SF2_2, and SF2_3 indicate the candidate 2-item pairs. SF2_3* is the candidate pair we selected. PROMIS IDs are: SF2_1 = PFC12, PFB7; SF2_2 = PFC12, PFC11; SF2_3 = PFC12, PFB13. Because SF2_1 as well as the SF1 have a shorter range, their results at the T-score threshold of 30 are not available.


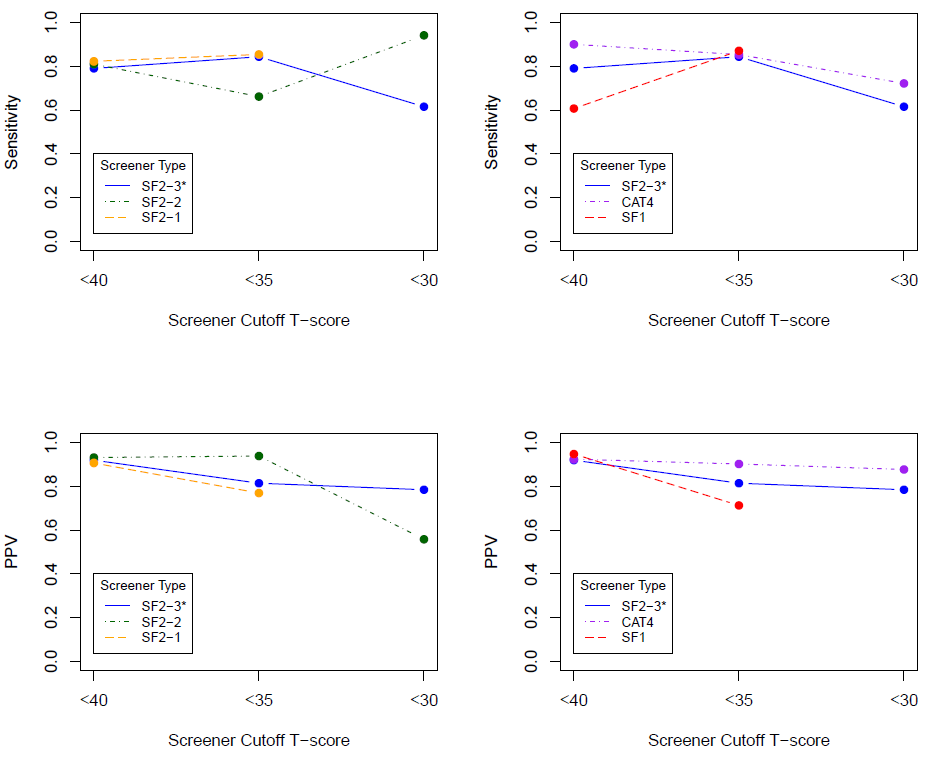

Supplement: Supplementary file 1 — Supplementary Material 1 [file 11136_2025_4015_MOESM1_ESM.docx]
