# Supplementary material for: Putting CATs and item banks to work: How to construct predictive and sensitive PROMIS screeners for use in ambulatory oncology
Source: Qual Life Res. 2025 Jul 26;34(10):2775–85. doi: 10.1007/s11136-025-04015-9 (PMC12535503; doi:10.1007/s11136-025-04015-9)
Supplement: Supplementary file 2 — Supplementary Material 2 [file 11136_2025_4015_MOESM2_ESM.docx]

Table S1. Inclusion and Exclusion Decisions of PROMIS Screener Candidate Items after Identification by CAT simulations

Note: “High Inf at threshold” (1) or (2) refers to the first or second highest level of information weighted by the threshold distribution. These are normal distributions defined as T-score = 60 (all domains except Physical Function, which is 35), and SD = 10. Item IDs are PROMIS IDs.

|  | **Item ID** | **Included/Excluded as pair for diagnostic testing** | **Notes** |  |  |  |
| --- | --- | --- | --- | --- | --- | --- |
| Anxiety | EDANX53 | Included | First item in CAT, included in all pairs tested | | | |
|  | EDANX41 | Included | Selected for final screener, High Inf at threshold (2) | | | |
|  | EDANX40 | Included | High Inf at threshold (1) | | |  |
|  | EDANX54 | Included |  |  |  |  |
|  | EDDEP30 | Excluded, similar to EDANX41 (worry) |  |  |  |  |
|  | EDANX46 | Excluded, similar to EDANX54 (nervous, tense) |  |  |  |  |
| Depression | EDDEP29 | Included | First item in CAT, included in all pairs tested | | | |
|  | EDDEP04 | Included | Selected for final screener, High Inf at threshold (2) | | | |
|  | EDDEP41 | Included | High Inf at threshold (1) | | |  |
|  | EDDEP36 | Included |  |  |  |  |
|  | EDDEP17 | Excluded, similar to EDDEP29 (sad and depressed) |  |  |  |  |
|  | EDDEP22 | Excluded, similar to EDDEP22 (failure and worthless) |  |  |  |  |
| Pain Interference | PAININ9 | Included | First item in CAT, incl. in all pairs tested, High Inf threshold ( | | | |
|  |  |  | at threshold (1) | | |  |
|  | PAININ10 | Included | Selected for final screener | | |  |
|  | PAININ31 | Included | High inf at threshold (2) | | | |
|  | PAININ3 | Excluded, overlaps with depression (enjoyment of life) |  |  |  |  |
|  | PAININ22 | Excluded, too narrow (house work) |  |  |  |  |
|  | PAININ26 | Excluded, similar to PAININ31 (social activities) |  |  |  |  |
|  | PAININ36 | Excluded, similar to PAININ31 (social activities) |  |  |  |  |
| Fatigue | FATIMP3 | Included | First item in CAT, incl. in all pairs tested, High Inf at | | | |
|  |  |  | at threshold (1) | | |  |
|  | AN3 | Included | Selected for final screener, High Inf at | | |  |
|  |  |  | threshold (2) |  |  |  |
|  | HI7 | Included |  |  |  |  |
|  | FATEXP40 | Included |  |  |  |  |
|  | FATEXP41 | Excluded, similar to FATEXP40 (average fatigue) |  |  |  |  |
|  | FATEXP20 | Excluded, similar to FATIMP3 (frequency formulation) |  |  |  |  |
|  | FATEXP35 | Excluded, similar to FATEXP40 (average fatigue) |  |  |  |  |
| Physical Function | PFC12 | Included | First item in CAT, included in all pairs tested | | | |
|  | PFB13 | Included | Selected for final screener | | |  |
|  | PFB7 | Included |  |  |  |  |
|  | PFC11 | Included | High Inf at threshold (1) | | | |
|  | PFA11 | Excluded, similar to PFC11 (yard work) | High Inf at threshold (2) | | |  |
|  | PFB5r1 | Excluded, too narrow and difficult (hiking hills) |  |  |  |  |
